# Two-Dimensional Fluorescence Lifetime Correlation Spectroscopy: Concepts and Applications

**DOI:** 10.3390/molecules23112972

**Published:** 2018-11-14

**Authors:** Takuhiro Otosu, Shoichi Yamaguchi

**Affiliations:** Department of Applied Chemistry, Graduate School of Science and Engineering, Saitama University, 255 Shimo-Okubo, Sakura, Saitama 338-8570, Japan; otosu@apc.saitama-u.ac.jp

**Keywords:** fluorescence correlation spectroscopy, fluorescence lifetime, single-molecule spectroscopy, conformational dynamics

## Abstract

We review the basic concepts and recent applications of two-dimensional fluorescence lifetime correlation spectroscopy (2D FLCS), which is the extension of fluorescence correlation spectroscopy (FCS) to analyze the correlation of fluorescence lifetime in addition to fluorescence intensity. Fluorescence lifetime is sensitive to the microenvironment and can be a “molecular ruler” when combined with FRET. Utilization of fluorescence lifetime in 2D FLCS thus enables us to quantify the inhomogeneity of the system and the interconversion dynamics among different species with a higher time resolution than other single-molecule techniques. Recent applications of 2D FLCS to various biological systems demonstrate that 2D FLCS is a unique and promising tool to quantitatively analyze the microsecond conformational dynamics of macromolecules at the single-molecule level.

## 1. Introduction

With the development of the confocal microscope, fluorescence correlation spectroscopy (FCS) has been widely applied to analyze the diffusion coefficients of molecules and their conformational dynamics [1,2,3,4]. FCS analyzes the correlation of single molecules through the temporal fluctuation of fluorescence signals that are detected from a tiny focal region of a microscope objective. Due to its simple experimental setup and wider applicable range of sample concentration compared to “strict” single-molecule experiments, FCS is becoming an indispensable tool not only in biology, but also in molecular science and physical chemistry [5,6,7,8]. Furthermore, the combination of FCS with various sophisticated microscope systems and the introduction of additional experimental parameters to FCS methodology makes FCS an advanced technique, beyond conventional analytical tools [9,10,11,12,13]. Here, we focus on how to incorporate fluorescence lifetime information in FCS.

In 2002, two groups independently published papers that were related to the utilization of fluorescence lifetime in FCS. Enderlein and his coworkers reported the concept and application of “Time-resolved FCS” [14], which is now recognized as Fluorescence Lifetime Correlation Spectroscopy (FLCS) [15,16]. In FLCS, one uses fluorescence lifetime information to calculate the filter functions of all species in a heterogeneous system. Utilization of filter functions enables us to extract the species-specific auto- and cross-correlation functions from the ensemble photon data. Since the first application of FLCS (time-resolved FCS at that time) to a dye mixture system [14], several applications have shown the usefulness of FLCS in various biological systems [17,18,19]. On the other hand, Yang and Xie built the conceptual basis of photon-by-photon analysis of excitation-detection delay time (fluorescence lifetime) [20,21], which was later extended and experimentally demonstrated by Ishii and Tahara as Lifetime-weighted FCS and Two-Dimensional Fluorescence Lifetime Correlation Spectroscopy (2D FLCS) [22,23,24,25]. In particular, 2D FLCS allows us to detect the inhomogeneity of the system and to quantitatively analyze the diffusion and the interconversion dynamics of all species with distinct lifetimes in a species-specific manner as described below. The excellent reviews of FLCS can be found in the literature [26,27,28,29]. However, a comprehensive review of 2D FLCS has not yet been published, except for a book [30]. Recently, applications of 2D FLCS to various biological systems come to reveal the high performance of 2D FLCS to quantify the microsecond dynamics of macromolecules at the single-molecule level. Therefore, we strongly believe that a handy but accurate review of 2D FLCS is now needed especially for potential users. In this paper, we review the basic concepts and recent applications of 2D FLCS, only focusing on essential points, without challenging mathematics or too many details.

## 2. Instrumentation of 2D FLCS

**Figure 1:**
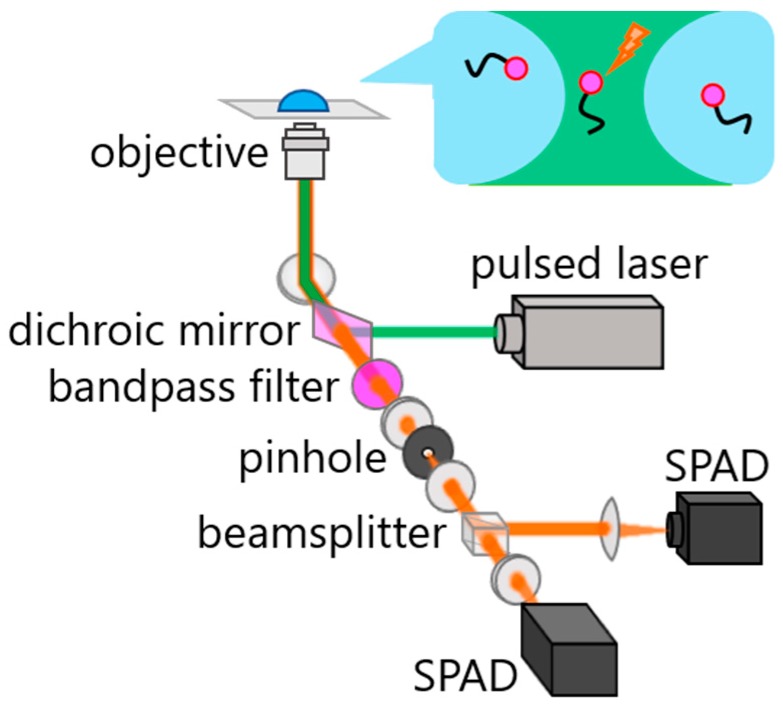
Schematic of a typical 2D FLCS instrument.

Figure 1 shows a typical optical setup of 2D FLCS. It is usually equipped with a confocal microscope, but is compatible with other microscope systems, e.g., a total-internal reflection microscope [31]. In addition to the ordinary confocal microscope system, 2D FLCS requires a pulsed laser, a single-photon detector (or two detectors), and a time-correlated single-photon counting (TCSPC) module implementing time-tagged (or time-tagged time-resolved) mode. Either a femtosecond Ti-sapphire laser or a picosecond diode laser is currently used as an excitation light source. The repetition rate used is typically 20–80 MHz, which gives a sufficient excitation-detection time window for typical fluorescent dyes. Laser is reflected by a dichroic mirror and directed into a microscope objective to excite the sample. Fluorescence emitted from the focal region is collected by the same objective with a back-scattering geometry. Rayleigh scattering and other unwanted background signals are separated from the fluorescence by passing through a dichroic mirror and a bandpass filter. The fluorescence is then passed through a pinhole to eliminate off-focus signals and is separated into two by using a nonpolarized 50/50 beamsplitter before being focused onto single-photon detectors. Usually, single-photon avalanche diodes (SPADs) are used for the detectors. There are two advantages to utilizing this two-detector system. One is the reduction of detection deadtime and the other is the elimination of the afterpulsing effect from the correlation data [32]. Afterpulse is a “fake” signal emitted from a SPAD with a delay time of~µs from a “true” photon detection event. Because the detected photon and the corresponding afterpulse is highly correlated, it affects the correlation data in the µs region in a one-detector system. However, a one-detector system also works well with a sophisticated method reported by Ishii and Tahara, which allows us to eliminate the afterpulsing effect from the FCS, as well as 2D FLCS data [33]. Electric signals from the detectors are sent to a TCSPC module, and temporal information of all detected photons is stored as photon data. Currently, either Harp series (PicoQuant) or SPC series (Becker & Hickl) can be used for the TCSPC module.

## 3. Analytical Method of 2D FLCS

2D FLCS analysis consists of three steps: (1) construction of a 2D emission-delay (microtime) correlation map, (2) subtraction of uncorrelated photon pairs, (3) conversion from the 2D emission-delay correlation map to a 2D lifetime correlation map. In this section, we briefly describe the procedure of each step. A more detailed description of each step can be found in the literature [23,24,25].

### 3.1. Construction of a 2D Emission-Delay Correlation Map

**Figure 2:**
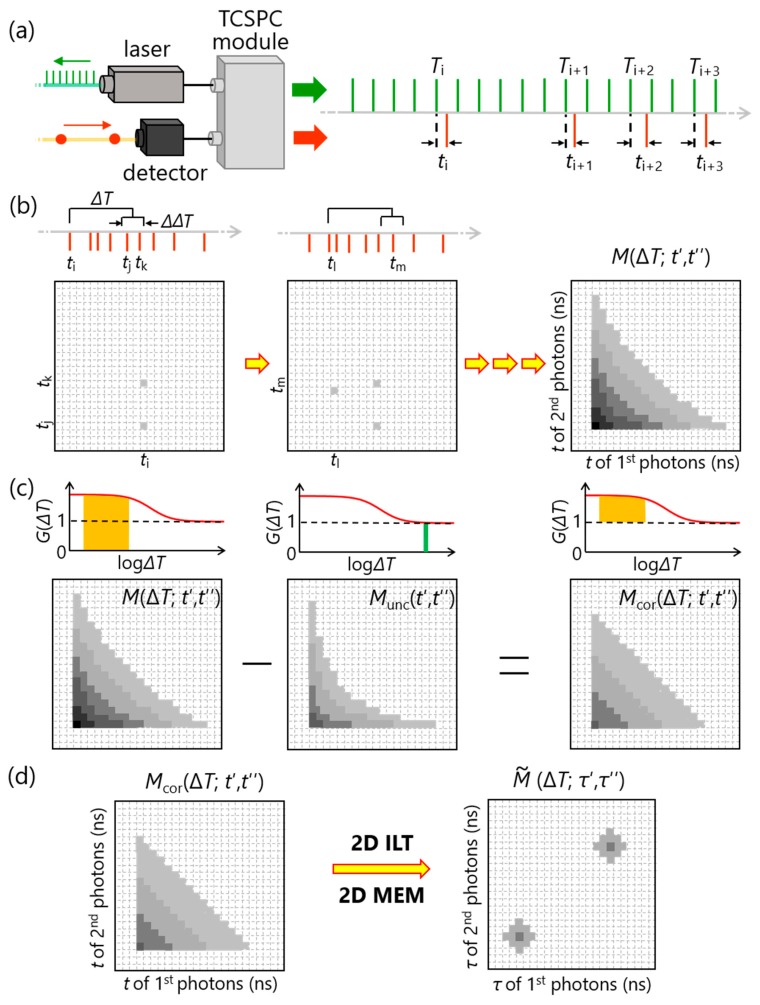
Schematic of 2D FLCS analysis. (**a**) Accumulation of photon data. For each detected photon, macrotime (*T*) and microtime (*t*) are stored as photon data. (**b**) Construction of a 2D emission-delay correlation map. (**c**) Subtraction of uncorrelated photon pairs. (**d**) Conversion from the 2D emission-delay correlation map to a 2D lifetime correlation map by 2D inverse Laplace transform (ILT) with the help of 2D maximum entropy method (2D MEM).

By using the 2D FLCS instrument shown in Figure 1, two pieces of temporal information can be obtained for each fluorescence photon, that is, macrotime and microtime (Figure 2a). Macrotime (*T*) is the detection time of the photon from the start of the experiment. Because the timescale of *T* (typically sub-µs~s) is much longer than the excitation-emission delay time (ps~ns), *T* is usually recorded by referring to the external clock that is given from a pulsed laser as synchronous signals. On the other hand, microtime (*t*) is the relative delay time of the photon detection with respect to the corresponding excitation pulse. This *t* is calculated on a TCSPC module by referring to the synchronous signals from the laser. This information of *T* and *t* is utilized to construct a 2D emission-delay correlation map.

**Figure 3:**
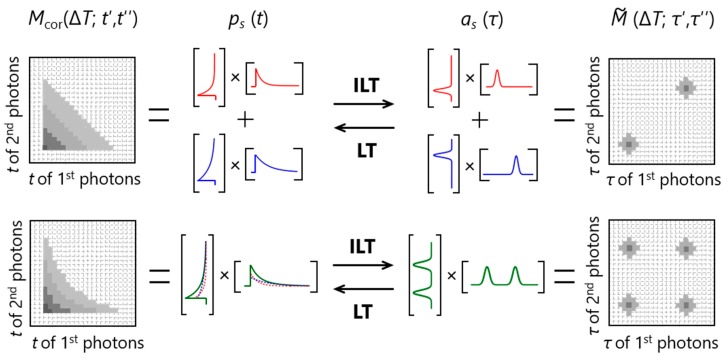
Relationships among a 2D emission-delay correlation map (*M*_cor_ (Δ*T*; *t*′, *t*′′)), a 2D lifetime correlation map ($\tilde{M}(\Delta T;\tau^{\prime },\tau^{\prime \prime })$), and the fluorescence decay curves (*p_s_* (*t*)) and lifetime distributions (*a_s_* (*τ*)) of independent species. ILT and LT denote inverse Laplace transform and Laplace transform, respectively.

In constructing a 2D emission-delay correlation map, one searches photon pairs which have the time interval of Δ*T* − ΔΔ*T*/2~Δ*T +* ΔΔ*T*/2 between the two photons, where ΔΔ*T* is an arbitrary macrotime window that is typically set to be ΔΔ*T* < Δ*T* (Figure 2b). Then, the microtime of the photons in the photon pair is plotted into a map. In this map, the horizontal axis corresponds to the microtime of the 1^st^ photons in the photon pair and the vertical axis corresponds to the photons detected after Δ*T ±* ΔΔ*T*/2 from the 1st photons (denoted as 2nd photons in Figure 2 and Figure 3). By repeating this procedure for all detected photons, one can get a 2D emission-delay (microtime) correlation map (*M* (Δ*T*; *t*′, *t*″)).

### 3.2. Subtraction of Uncorrelated Photon Pairs

The constructed 2D emission-delay correlation map (*M* (Δ*T*; *t*′, *t*″)) contains a contribution from uncorrelated photon pairs, in addition to that from correlated pairs emitted by single molecules. Uncorrelated photon pairs include (1) photon pairs in which each photon is emitted from a different molecule, and (2) photon pairs in which one or both photons stem from background signals. A key characteristic of such uncorrelated photon pairs is that those photon pairs are independent of Δ*T* [23], which can be utilized to subtract the contribution of the uncorrelated photon pairs from *M* (Δ*T*; *t*′, *t*″) to obtain a 2D emission-delay correlation map of single molecules. Figure 2c shows a schematic of this procedure. The constructed 2D emission-delay correlation map at Δ*T* corresponds to the ordinary intensity-correlation amplitude from 0 to *G* (Δ*T*). To subtract the uncorrelated contribution, one also constructs a 2D emission-delay correlation map at longer Δ*T* that is much longer than the average residence time of the molecules in a focal region. Because the correlation is practically lost at that Δ*T*, the obtained map represents the uncorrelated photon pairs (*M*_unc_ (*t*′, *t*″)) corresponding to the intensity-correlation amplitude from 0 to 1. Thus, *M*_unc_ (*t*′, *t*″) can be used to obtain the correlated map:

(1) $$M_{\mathrm{cor}}(\Delta T;t^{\prime },t^{\prime \prime })=M(\Delta T;t^{\prime },t^{\prime \prime })-M_{\mathrm{unc}}(t^{\prime },t^{\prime \prime }).$$

Because the resulting correlated map (*M*_cor_ (Δ*T*; *t*′, *t*′′)) only contains the single-molecule correlation, the information in this map is equivalent to the data obtained with a “strict” single-molecule experiment. Furthermore, the time resolution of FCS is superior to such (strict) single-molecule spectroscopy. Therefore, 2D FLCS can analyze rapid interconversion dynamics at the single-molecule level with a microsecond time resolution, which is the main advantage of 2D FLCS beyond the ordinary single-molecule spectroscopy.

### 3.3. Inverse Laplace Transform with the Help of 2D Maximum Entropy Method

Even though a 2D emission-delay correlation map of single molecules (*M*_cor_ (Δ*T*; *t*′, *t*″)) is successfully extracted by step 2, it is still difficult to interpret the map directly from its appearance. To further analyze this map, 2D inverse Laplace transform (2D ILT) helps us to convert this map to a 2D lifetime correlation map ($\tilde{M}(\Delta T;\tau^{\prime },\tau^{\prime \prime })$) (Figure 2d). Unfortunately, it is well known that ILT is numerically unstable. To suppress the numerical instability, 2D maximum entropy method (MEM) is employed in 2D FLCS.

Because *M*_cor_ (Δ*T*; *t*′, *t*″) is described with the sum of the single-molecule correlation of all emitted species, it can be represented by the following equations:

(2) $$M_{\mathrm{cor}}\left(\Delta T;{t^{\prime }}_{i},{t^{\prime \prime }}_{j}\right)=\sum_{k=1}^{L}\sum_{l=1}^{L}\tilde{M}\left(\Delta T;{\tau^{\prime }}_{k},{\tau^{\prime \prime }}_{l}\right)\exp(-{t^{\prime }}_{i}/{\tau^{\prime }}_{k})\exp(-{t^{\prime \prime }}_{j}/{\tau^{\prime }}_{l}),$$

(3) $$\tilde{M}\left(\Delta T;{\tau^{\prime }}_{k},{\tau^{\prime \prime }}_{l}\right)=\sum_{s=1}^{n}a_{s}({\tau^{\prime }}_{k})a_{s}({\tau^{\prime \prime }}_{l}),$$

where *L* is the number of data points along the lifetime (*τ*) scale, *a_s_* (*τ*) is the independent lifetime distribution of species *s*, and *n* is the number of the independent species. In 2D MEM analysis, one prepares a trial 2D lifetime correlation map (${\tilde{M}}^{0}\left(\Delta T;\tau^{\prime },\tau^{\prime \prime }\right)$) to calculate a trial 2D emission-delay correlation map ($M_{\mathrm{cor}}^{0}\left(\Delta T;t^{\prime },t^{\prime \prime }\right)$) and compare it with an experimental one.

(4) $$M_{\mathrm{cor}}^{0}\left(\Delta T;{t^{\prime }}_{i},{t^{\prime \prime }}_{j}\right)=\sum_{k=1}^{L}\sum_{l=1}^{L}{\tilde{M}}^{0}\left(\Delta T;{\tau^{\prime }}_{k},{\tau^{\prime \prime }}_{l}\right)\exp(-{t^{\prime }}_{i}/{\tau^{\prime }}_{k})\exp(-{t^{\prime \prime }}_{j}/{\tau^{\prime \prime }}_{l}),$$

(5) $${\tilde{M}}^{0}\left(\Delta T;{\tau^{\prime }}_{k},{\tau^{\prime \prime }}_{l}\right)=\sum_{s=1}^{n}a_{s}^{0}({\tau^{\prime }}_{k})a_{s}^{0}({\tau^{\prime \prime }}_{l})$$

The agreement between $M_{\mathrm{cor}}\left(\Delta T;t^{\prime },t^{\prime \prime }\right)$ and $M_{\mathrm{cor}}^{0}\left(\Delta T;t^{\prime },t^{\prime \prime }\right)$ is evaluated based on the chi-square value (*χ*^2^):

(6) $$\chi^{2}=\frac{1}{K^{2}-1}\sum_{i=1}^{K}\sum_{j=1}^{K}\frac{{\left\{M_{\mathrm{cor}}^{0}\left(\Delta T;{t^{\prime }}_{i},{t^{\prime \prime }}_{j}\right)-M_{\mathrm{cor}}\left(\Delta T;{t^{\prime }}_{i},{t^{\prime \prime }}_{j}\right)\right\}}^{2}}{M\left(\Delta T;{t^{\prime }}_{i},{t^{\prime \prime }}_{j}\right)},$$

where *K* is the maximum microtime channel. Moreover, the entropy (*S*) of ${\tilde{M}}^{0}\left(\Delta T;\tau^{\prime },\tau^{\prime \prime }\right)$ can be defined as [34]:

(7) $$S=\sum_{s=1}^{n}\sum_{k=1}^{L}\left\{a_{s}^{0}\left(\tau_{k}\right)-m_{s}\left(\tau_{k}\right)-a_{s}^{0}\left(\tau_{k}\right)\ln\frac{a_{s}^{0}\left(\tau_{k}\right)}{m_{s}^{}\left(\tau_{k}\right)}\right\}.$$

In Equation (7), $m_{s}\left(\tau \right)$ is a prior knowledge of $a_{s}^{0}\left(\tau \right)$ and acts as a bias for *S*. Usually, one sets a constant value for $m_{s}\left(\tau \right)$ (no bias). The optimum ${\tilde{M}}^{0}\left(\Delta T;\tau^{\prime },\tau^{\prime \prime }\right)$ that minimizes the following *Q* value is then searched and determined,

(8) $$Q=\chi^{2}-\frac{2S}{\eta },$$

where *η* is the regularizing constant. Because the number of independent species is unknown, 2D MEM analysis is initially performed with small *n* (typically 1 or 2) and *n* is increased until the simulated 2D emission-delay correlation map reproduces the experimental one, as judged from *χ*^2^ and the residuals. It is noted that one can also perform global 2D MEM analysis for several 2D emission-delay correlation maps at different Δ*T*s, which assures a more reliable and stable conversion to the corresponding 2D lifetime correlation maps. Analytical details of global 2D MEM analysis are elaborated in [25].

On the basis of the procedure described above, one can analyze the number of independent lifetime species and their fluorescence lifetime distributions. Furthermore, the fluorescence decay curve of each independent species (*p_s_* (*t*)) can be obtained by performing Laplace transform (LT) on the corresponding *a_s_* (*τ*). The relationships among *M*_cor_ (Δ*T*; *t*′, *t*′′), $\tilde{M}(\Delta T;\tau^{\prime },\tau^{\prime \prime })$, *p_s_* (*t*) and *a_s_* (*τ*) are summarized in Figure 3. In the upper panel, two diagonal peaks appear in $\tilde{M}(\Delta T;\tau^{\prime },\tau^{\prime \prime })$. This peak pattern is a representative of the existence of two independent species and the position of each diagonal peak represents the lifetime of each species as observed in the corresponding *a_s_* (*τ*). On the other hand, two off-diagonal peaks in addition to the diagonal peaks appear in $\tilde{M}(\Delta T;\tau^{\prime },\tau^{\prime \prime })$ in the lower panel. This represents the existence of two independent species that are interconverted with each other with the timescale faster than Δ*T*. In this case, two species with distinct lifetimes are indistinguishable at this Δ*T*, so that one independent lifetime distribution with two peaks and a double-exponential decay curve is observed in *a_s_* (*τ*) and *p_s_* (*t*), respectively. Thus, the two examples in Figure 3 indicate that the emergence of off-diagonal peaks with increasing Δ*T* (change from the upper to lower examples in Figure 3) tells us the timescale of interconversion between the corresponding species.

## 4. Application of 2D FLCS

**Figure 4:**
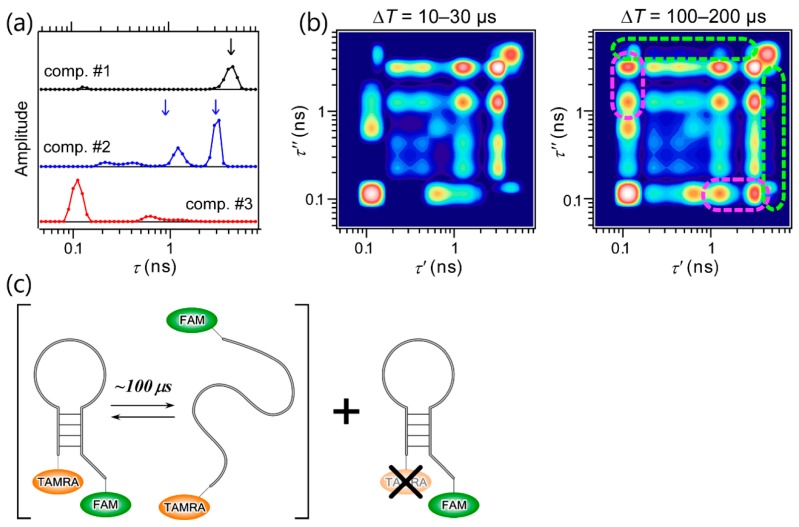
2D FLCS of DNA hairpin. (**a**) Three independent lifetime distributions observed at Δ*T* = 10–30 µs, (**b**) 2D lifetime correlation maps at Δ*T* = 10–30 and 100–200 µs. Dashed lines indicate the regions where the cross peaks between components 1 and 2, 3 (green) and those between components 2 and 3 (magenta) are expected to appear. (**c**) Sketch of the folding dynamics of the DNA hairpin observed by 2D FLCS. Adapted with permission from Ref. [25], Copyright 2013, American Chemical Society.

The most important feature of 2D FLCS is that it enables us to observe the conformational dynamics of macromolecules with high time resolution through the time evolution of 2D lifetime correlation maps; specifically, the emergence of off-diagonal peaks. This was first demonstrated by Ishii and Tahara by performing 2D FLCS on the conformational dynamics of a DNA hairpin [25]. DNA hairpin is a single-chain DNA consisting of stem and loop regions, and base pairs are formed on the stem region. The stability of the stem region highly depends on the solution conditions such as the ionic strength. Thus, the closed (where the base pairs are formed on the stem region) and open forms (where the base pairs dissociate to form single-stranded conformation) of DNA coexist in equilibrium under a certain condition. To analyze the conformational dynamics between the closed and open forms by 2D FLCS, two fluorophores, 6-carboxyfluorescein (FAM) and tetramethylrhodamine (TAMRA), are attached on 5′- and 3′-terminals of the DNA hairpin, respectively (Figure 4c). Because the fluorescence spectrum of FAM overlaps with the absorption spectrum of TAMRA, the fluorescence lifetime of FAM is sensitive to the FRET efficiency, that is, the end-to-end distance of the DNA. Therefore, closed-open conformational dynamics can be analyzed by 2D FLCS through the correlation of the conformation-dependent fluorescence lifetime of FAM (FRET donor).

Figure 4a shows three independent lifetime distributions observed at Δ*T* = 10–30 µs, and the 2D lifetime correlation maps at Δ*T* = 10–30 and 100–200 µs are shown in Figure 4b. Remarkably, a single lifetime peak in component 1 matches the fluorescence lifetime of similar DNA without an acceptor dye (black arrow in Figure 4a), and two major peaks in component 2 are nearly identical to those observed in the data of a single-stranded DNA without base pairs (blue arrows in Figure 4a). Based on this agreement, components 1 and 2 are confidently assigned to the acceptor-missing and the open form of the DNA hairpin, respectively. Component 3 is hence assigned to the closed form. The existence of the two peaks in component 2 suggests that the open form of the DNA hairpin is highly flexible, and the exchange between different conformations corresponding to the two peaks occurs faster than 10 µs. Furthermore, the closed-open dynamics of the DNA hairpin is clearly seen in the 2D lifetime correlation maps, even though these look complex due to several major and minor peaks in each component. In the map at Δ*T* = 100–200 µs, off-diagonal peaks between components 2 and 3 are clearly seen that are absent in the map at Δ*T* = 10–30 µs, the regions of which are marked with magenta (Figure 4b). On the other hand, no off-diagonal peaks between component 1 and other components are observed in the map (green dashed region). This is reasonable because the fluorescence lifetime of the donor in the acceptor-missing DNA is insensitive to the closed-open conformational transition. On the basis of the reliable assignment of the three independent lifetime distributions, the observation of the emergence of the off-diagonal peaks between components 2 and 3 and the application of lifetime-weighted FCS, another useful variant of FCS utilizing fluorescence lifetime information [22,25], it was concluded that the closed-open conformational transition of the DNA hairpin occurs with the time scale of ~100 µs.

The high time resolution of 2D FLCS was further verified by applying it to the study of protein folding. Protein folding is a hierarchical process involving the initial conformational collapse, the formation of secondary structure and the final tertiary-structure formation. The timescale of each conformational transition ranges from sub-microseconds to seconds or even hours. In particular, sub-µs and µs conformational dynamics of proteins have attracted much attention recently, because the advancement of MD simulation now enables us to simulate the atomic details of protein dynamics up to µs–ms timescale [35,36]. However, the applications of the ordinary single-molecule technique are restricted to the dynamics up to ~100 µs due to the limitation of available photon number. Thus, the quantification of the microsecond dynamics is still a challenging task.

**Figure 5:**
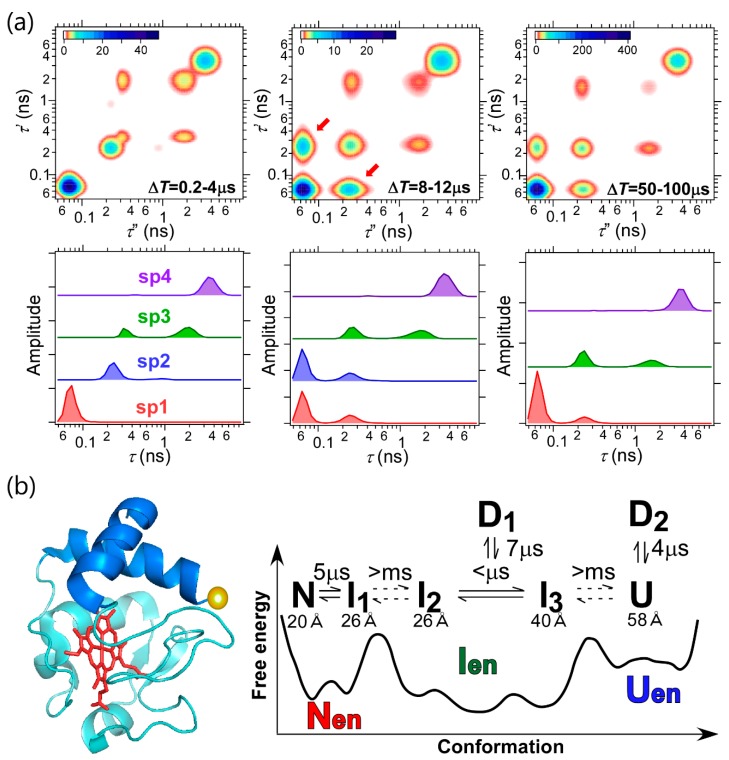
2D FLCS of cytochrome *c*. (**a**) 2D lifetime correlation maps at Δ*T* = 0.2–4, 8–12, and 50–100 µs (upper) and the corresponding independent lifetime distributions (lower). (**b**) (left) Native-state structure of cytochrome *c* (PDB ID:1YCC). N- and C-terminal helices are highlighted with blue. The position of the donor dye is shown by a yellow sphere. (right) Schematic free-energy landscape and the relevant conformational dynamics of cytochrome *c* at pH 3.5. The equilibration times among conformers and the donor–heme distances evaluated in the present work are given. Adapted with permission from Macmillan Publishers Ltd.: Nature Communications Ref. [37], Copyright 2015.

To elucidate the microsecond dynamics of proteins, 2D FLCS was applied to cytochrome *c* (cyt *c*) (Figure 5) [37]. Cyt *c* is a heme protein and has been widely used as a model protein for protein folding [38,39,40,41,42,43]. To study the conformational transition of this protein, a donor dye, Alexa 546, was attached to the single free cysteine residue located in the C-terminal region (Figure 5b). Because the fluorescence spectrum of this dye overlaps with the visible absorption band of heme, it is expected that the conformational transition of cyt *c* can be analyzed through the change in the fluorescence lifetime of the donor. 2D FLCS was performed at pH 3.5, where the µs correlation decay corresponding to some conformational dynamics of cyt *c* was previously suggested by conventional FCS [42]. Figure 5a shows the 2D lifetime correlation maps and the corresponding lifetime distributions at Δ*T* = 0.2–4, 8–12, and 50–100 µs. At Δ*T* = 0.2–4 µs, four independent lifetime species are observed and one species (sp3) shows two peaks in the corresponding lifetime distribution. This suggests that five conformers of cyt *c* coexist at pH 3.5 and two of them are interconverted with each other with the timescale faster than ~1 µs. Furthermore, the emergence of off-diagonal peaks between sp1 and sp2 (indicated by arrows in Figure 5a) is clearly observed in the 2D lifetime correlation maps at Δ*T* = 8–12 and 50–100 µs. Because the shortest lifetime species can be assigned to the native state of cyt *c* due to its compact structural nature (high FRET efficiency), the observation of these off-diagonal peaks indicates that the conformational transition between the native state (sp1) and one of the intermediate states (sp2) occurs with the timescale at ~5 µs. As mentioned, spontaneous conformational dynamics at several microseconds is hardly quantified with conventional single-molecule spectroscopy. Therefore, this study unequivocally demonstrated the high performance of 2D FLCS to elucidate the rapid conformational dynamics of proteins at the single-molecule level. Further 2D FLCS analysis in this study gave the complex folding scheme of cyt *c* including seven conformers (Figure 5b).

**Figure 6:**
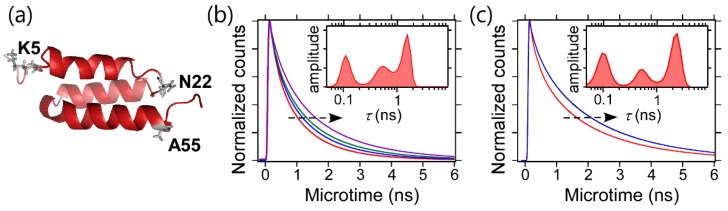
(**a**) NMR structure of B domain of protein A (PDB ID: 1SS1). The residues that were mutated to cysteine are shown by sticks. (**b**) Fluorescence decay curves of the FRET donor in the native state of K5C/Y15F/A55C mutant of BdpA at 1.0 (red), 2.0 (blue), 2.5 (green), and 3.0 M (purple) of guanidium hydrochloride (GdmCl). The corresponding independent lifetime distribution at 2.0 M GdmCl is also shown in the figure. (**c**) Fluorescence decay curves of the FRET donor in the unfolded state of Y15F/N22C/A55C mutant of BdpA at 2.0 (red) and 4.0 M (blue) of GdmCl. The corresponding independent lifetime distribution at 2.0 M GdmCl is also shown in the figure. In (**b**,**c**), these decay curves were obtained by performing a global 2D MEM analysis on the 2D emission-delay correlation maps at Δ*T* = 1–20, 50–100, and 400–600 µs. The arrows in (**b**,**c**) represent the shift of fluorescence decay curves with increasing GdmCl concentration. Adapted with permission from Ref. [44] Copyright 2017, American Chemical Society.

The application to cyt *c* revealed the high time resolution of 2D FLCS to detect the microsecond dynamics of proteins. In addition to the time resolution, 2D FLCS gives us the valuable structural information through the lifetime distribution and the corresponding fluorescence decay curve of each conformer. This is well demonstrated by applying it to the study of B domain of protein A (BdpA) [44]. BdpA is a small, single-domain globule protein which has three helices in its structure (Figure 6a) [45]. BdpA has also been utilized as a model protein for protein folding [46,47,48]. However, the native-state conformation of BdpA is still under debate [49,50,51]. To elucidate the conformational property of the native as well as the unfolded states of BdpA, 2D FLCS was applied. In this study, two FRET mutants of BdpA were prepared, that is, K5C/Y15F/A55C mutant and Y15F/N22C/A55C mutant, and FRET dyes were attached to the cysteine residues. In K5C/Y15F/A55C mutant, FRET pair is located at the both ends of BdpA. Thus, the donor fluorescence of this mutant is sensitive to the overall conformational change in the native state but is insensitive to the unfolded conformation due to the longer donor-acceptor distance. Indeed, 2D FLCS of this mutant revealed two independent species and the fluorescence lifetime of longer-lifetime species was comparable to that of a free donor dye and insensitive to the denaturant concentration. Therefore, we assigned the longer-lifetime species to the unfolded state, and the native-state conformation was analyzed based on the lifetime distribution and the corresponding fluorescence decay curve of the shorter lifetime species. In Y15F/N22C/A55C mutant, the FRET pair are located close to each other in the native state so that the donor fluorescence of the native state is hardly observed in this mutant. On the other hand, this mutant is suitable to analyze the conformational property of the unfolded state. Therefore, the usage of the two FRET mutants gives us the opportunity to examine the conformational properties of the native (from K5C/Y15F/A55C mutant) and unfolded (from K5C/Y15F/A55C mutant) states of BdpA.

The lifetime distributions of the FRET donor in the native (Figure 6b) and unfolded (Figure 6c) states of BdpA obtained by 2D FLCS with 10 µs time resolution show multiple lifetime peaks, suggesting that both the native and unfolded states are inhomogeneous and multiple conformers are interconverted with each other with the timescale faster than 10 µs. Furthermore, independent fluorescence decay curves of both states obtained by Laplace transform of the corresponding lifetime distributions shift to longer lifetime sides with increasing the denaturant concentration (arrows in Figure 6b,c). This clearly shows that the native-state ensemble in addition to that of the unfolded state are highly sensitive to the denaturant condition. The ensemble nature and denaturant sensitivity of the native-state conformation of BdpA are surprising, because a native state was believed to have a solid conformation. Based on the results of the FRET mutants, it was suggested that the fraying of N-terminal helix is the origin of the ensemble nature of the native-state conformation in BdpA. Because a FRET donor in both the native and unfolded states shows multi-exponential decay kinetics and the fluorescence lifetimes of both states are sensitive to the denaturant condition, it is hard to analyze the fluorescence decay curve (lifetime) of each state based on the ensemble-averaged experiments, e.g., the global analysis of the denaturant-dependent fluorescence decay curves. Thus, the plasticity of the native-state conformation (ensemble nature of the native state) and the rapid interconversions in the native-state ensemble can only be verified with the high time resolution and structural sensitivity of 2D FLCS.

## 5. Future Perspectives

2D FLCS utilizes fluorescence lifetime to analyze the diffusion and rapid conformational dynamics of macromolecules with the high time resolution and structural sensitivity. Furthermore, it can be achieved with a simple experimental configuration as shown in Figure 1. Therefore, this technique is compatible with various optical systems where fluorescence lifetime can be measured (e.g., laser scanning microscopy [52]), and can also be combined with other fluorescence lifetime-based analytical methods (e.g., phasor plot analysis [53]). In particular, the combination of 2D FLCS with FLCS will be a promising tool for the future application of 2D FLCS. FLCS developed by Enderlein utilizes fluorescence lifetime to extract the species-specific auto-and cross-correlation curves from ensemble photon data as described in the Introduction [15,16]. Despite its simple analytical procedure, FLCS has one requirement that fluorescence decay curves (lifetimes) of all species in a system have to be known in advance. This requirement may be satisfied only when the suitable reference data is available. In the case of the folding intermediate states of proteins, for example, these is no a priori knowledge of fluorescence lifetimes. Thus, this requirement often limits the application of FLCS. However, this limitation can be overcome by the combination with 2D FLCS. Specifically, determination of independent lifetime distributions of all species by 2D FLCS, their conversions to the corresponding fluorescence decay curves by LT, and the application of these decay curves to FLCS analysis not only remove the limitation of FLCS but also broaden the application of fluorescence lifetime-correlation analysis to various inhomogeneous and complex systems. Actually, this attempt has recently been reported by the Tahara group and us [31,54].

Very recently, we also applied this methodology to examine the diffusion of lipids in a supported lipid bilayer (SLB) [55]. SLB is a model biomembrane formed on a solid support such as a glass [56]. Besides its flatness and simple preparation procedure, the advantage of utilizing SLB is its fluidity, which enables us to examine the dynamical property of a lipid membrane such as flip-flop kinetics of lipids [57]. Despite the wider applications of SLB, however, the effect of a solid support on the diffusion dynamics of lipids in the two leaflets (monolayers) of SLB, that is, the proximal (facing a solid support) and distal (facing bulk solution) leaflets, is still controversial [58,59,60]. We then performed 2D FLCS and FLCS on SLB to elucidate the diffusion of lipids in both leaflets of SLB. To analyze the diffusion of lipids in each leaflet independently, potassium iodide, a famous fluorescence quencher, was added to the bulk solution above SLB, by which the fluorescence lifetime of lipids in the distal leaflet becomes shorter than that in the proximal leaflet. Leaflet-specific autocorrelation curves calculated by the combination of 2D FLCS and FLCS clearly showed that the diffusion of lipids in the proximal leaflet of SLB is substantially slower than that in the distal leaflet due to the stronger interaction between the lipids in the proximal leaflet and a glass surface. Because leaflet-selective diffusion analysis is difficult with the conventional FCS, this demonstrates that the combination of 2D FLCS and FLCS will be a standard tool to explore the complex biological systems.

## 6. Conclusions

FCS has long been utilized to analyze the diffusion and rapid conformational dynamics of macromolecules. The high time resolution (ns–µs) of FCS beyond conventional single-molecule spectroscopy has attracted our attention with the advancement of all-atom MD simulation. However, the fluorescence intensity-based correlation analysis has a limitation to elucidate the origin of the fluorescence fluctuation, where only model-dependent fitting analysis is possible. 2D FLCS, on the other hand, enables us to quantitatively analyze the rapid conformational dynamics of macromolecules in a model-free manner though the correlation of fluorescence lifetime without sacrificing the high time resolution of FCS. Thus, future 2D FLCS will open the way to elucidate the complex, ensemble nature of macromolecule conformations and their significance in various biological functions.

## References

[1] Elson E.L., Magde D. (1974). Fluorescence correlation spectroscopy. I. Conceptual basis and theory. Biopolymers.

[2] Dertinger T., Pacheco V., von der Hocht I., Hartmann R., Gregor I., Enderlein J. (2007). Two-focus fluorescence correlation spectroscopy: A new tool for accurate and absolute diffusion measurements. Chemphyschem.

[3] Nettels D., Hoffmann A., Schuler B. (2008). Unfolded protein and peptide dynamics investigated with single-molecule fret and correlation spectroscopy from picoseconds to seconds. J. Phys. Chem. B.

[4] Elson E.L. (2011). Fluorescence correlation spectroscopy: Past, present, future. Biophys. J..

[5] Widengren J., Mets U., Rigler R. (1995). Fluorescence correlation spectroscopy of triplet-states in solution: A theoretical and experimental study. J. Phys. Chem..

[6] Soranno A., Buchli B., Nettels D., Cheng R.R., Muller-Spath S., Pfeil S.H., Hoffmann A., Lipman E.A., Makarov D.E., Schuler B. (2012). Quantifying internal friction in unfolded and intrinsically disordered proteins with single-molecule spectroscopy. Pros. Natl. Acad. Sci. USA.

[7] Krieger J.W., Singh A.P., Bag N., Garbe C.S., Saunders T.E., Langowski J., Wohland T. (2015). Imaging fluorescence (cross-) correlation spectroscopy in live cells and organisms. Nat. Protoc..

[8] Mikuni S., Yamamoto J., Horio T., Kinjo M. (2017). Negative correlation between the diffusion coefficient and transcriptional activity of the glucocorticoid receptor. Int. J. Mol. Sci..

[9] Bacia K., Kim S.A., Schwille P. (2006). Fluorescence cross-correlation spectroscopy in living cells. Nat. Methods.

[10] Hanson K.M., Davis S.K., Bardeen C.J. (2007). Two-photon standing-wave fluorescence correlation spectroscopy. Opt. Lett..

[11] Mueller V., Honigmann A., Ringemann C., Medda R., Schwarzmann G., Eggeling C. (2013). FCS in STED microscopy: Studying the nanoscale of lipid membrane dynamics. Method Enzymol..

[12] Benda A., Kapusta P., Hof M., Gaus K. (2014). Fluorescence spectral correlation spectroscopy (FSCS) for probes with highly overlapping emission spectra. Opt. Express.

[13] Vicidomini G., Ta H., Honigmann A., Mueller V., Clausen M.P., Waithe D., Galiani S., Sezgin E., Diaspro A., Hell S.W. (2015). STED-FLCS: An advanced tool to reveal spatiotemporal heterogeneity of molecular membrane dynamics. Nano Lett..

[14] Bohmer M., Wahl M., Rahn H.J., Erdmann R., Enderlein J. (2002). Time-resolved fluorescence correlation spectroscopy. Chem. Phys. Lett..

[15] Enderlein J., Gregor I. (2005). Using fluorescence lifetime for discriminating detector afterpulsing in fluorescence correlation spectroscopy. Rev. Sci. Instrum..

[16] Kapusta P., Wahl M., Benda A., Hof M., Enderlein J. (2007). Fluorescence lifetime correlation spectroscopy. J. Fluoresc..

[17] Humpolickova J., Beranova L., Stepanek M., Benda A., Prochazka K., Hof M. (2008). Fluorescence lifetime correlation spectroscopy reveals compaction mechanism of 10 and 49 kbp DNA and differences between polycation and cationic surfactant. J. Phys. Chem. B.

[18] Felekyan S., Kalinin S., Sanabria H., Valeri A., Seidel C.A.M. (2012). Filtered FCS: Species auto- and cross-correlation functions highlight binding and dynamics in biomolecules. Chemphyschem.

[19] Ghosh A., Isbaner S., Veiga-Gutierrez M., Gregor I., Enderlein J., Karedla N. (2017). Quantifying microsecond transition times using fluorescence lifetime correlation spectroscopy. J. Phys. Chem. Lett..

[20] Yang H., Xie X.S. (2002). Probing single-molecule dynamics photon by photon. J. Chem. Phys..

[21] Yang H., Xie X.S. (2002). Statistical approaches for probing single-molecule dynamics photon-by-photon. Chem. Phys..

[22] Ishii K., Tahara T. (2010). Resolving inhomogeneity using lifetime-weighted fluorescence correlation spectroscopy. J. Phys. Chem. B.

[23] Ishii K., Tahara T. (2012). Extracting decay curves of the correlated fluorescence photons measured in fluorescence correlation spectroscopy. Chem. Phys. Lett..

[24] Ishii K., Tahara T. (2013). Two-dimensional fluorescence lifetime correlation spectroscopy. 1. Principle. J. Phys. Chem. B.

[25] Ishii K., Tahara T. (2013). Two-dimensional fluorescence lifetime correlation spectroscopy. 2. Application. J. Phys. Chem. B.

[26] Kapusta P., Machan R., Benda A., Hof M. (2012). Fluorescence lifetime correlation spectroscopy (FLCS): Concepts, applications and outlook. Int. J. Mol. Sci..

[27] Machan R., Kapusta P., Hof M. (2014). Statistical filtering in fluorescence microscopy and fluorescence correlation spectroscopy. Anal. Bioanal. Chem..

[28] Basit H., Lopez S.G., Keyes T.E. (2014). Fluorescence correlation and lifetime correlation spectroscopy applied to the study of supported lipid bilayer models of the cell membrane. Methods.

[29] Ghosh A., Karedla N., Thiele J.C., Gregor I., Enderlein J. (2018). Fluorescence lifetime correlation spectroscopy: Basics and applications. Methods.

[30] Kapusta P.W.M., Erdmann R. (2015). Advanced Photon Counting: Applications, Methods, Instrumentation.

[31] Otosu T., Yamaguchi S. (2018). Total internal reflection two-dimensional fluorescence lifetime correlation spectroscopy. J. Phys. Chem. B.

[32] Burstyn H.C. (1980). Afterpulsing effects in photon-correlation experiments. Rev. Sci. Instrum..

[33] Ishii K., Tahara T. (2015). Correction of the afterpulsing effect in fluorescence correlation spectroscopy using time symmetry analysis. Opt. Express.

[34] Brochon J.C. (1994). Maximum-entropy method of data-analysis in time-resolved spectroscopy. Methods Enzymol..

[35] Lindorff-Larsen K., Piana S., Dror R.O., Shaw D.E. (2011). How fast-folding proteins fold. Science.

[36] Piana S., Lindorff-Larsen K., Shaw D.E. (2013). Atomic-level description of ubiquitin folding. Proc. Natl. Acad. Sci. USA.

[37] Otosu T., Ishii K., Tahara T. (2015). Microsecond protein dynamics observed at the single-molecule level. Nat. Commun..

[38] Goto Y., Hagihara Y., Hamada D., Hoshino M., Nishii I. (1993). Acid-induced unfolding and refolding transitions of cytochrome *c*: A three-state mechanism in H_2_O and D_2_O. Biochemistry.

[39] Bai Y.W., Sosnick T.R., Mayne L., Englander S.W. (1995). Protein-folding intermediates: Native-state hydrogen exchange. Science.

[40] Akiyama S., Takahashi S., Kimura T., Ishimori K., Morishima I., Nishikawa Y., Fujisawa T. (2002). Conformational landscape of cytochrome *c* folding studied by microsecond-resolved small-angle X-Ray scattering. Proc. Natl. Acad. Sci. USA.

[41] Perroud T.D., Bokoch M.P., Zare R.N. (2005). Cytochrome *c* conformations resolved by the photon counting histogram: Watching the alkaline transition with single-molecule sensitivity. Proc. Natl. Acad. Sci. USA.

[42] Werner J.H., Joggerst R., Dyer R.B., Goodwin P.M. (2006). A two-dimensional view of the folding energy landscape of cytochrome *c*. Proc. Natl. Acad. Sci. USA.

[43] Matsumoto S., Yane A., Nakashima S., Hashida M., Fujita M., Goto Y., Takahashi S. (2007). A rapid flow mixer with 11 µs mixing time microfabricated by a pulsed-laser ablation technique: Observation of a barrier-limited collapse in cytochrome *c* folding. J. Am. Chem. Soc..

[44] Otosu T., Ishii K., Oikawa H., Arai M., Takahashi S., Tahara T. (2017). Highly heterogeneous nature of the native and unfolded states of the B domain of protein a revealed by two-dimensional fluorescence lifetime correlation spectroscopy. J. Phys. Chem. B.

[45] Gouda H., Torigoe H., Saito A., Sato M., Arata Y., Shimada I. (1992). Three-dimensional solution structure of the B domain of staphylococcal protein a: Comparisons of the solution and crystal structures. Biochemistry.

[46] Bai Y.W., Karimi A., Dyson H.J., Wright P.E. (1997). Absence of a stable intermediate on the folding pathway of protein A. Protein Sci..

[47] Sato S., Religa T.L., Daggett V., Fersht A.R. (2004). Testing protein-folding simulations by experiment: B domain of protein A. Proc. Natl. Acad. Sci. USA.

[48] Huang F., Lerner E., Sato S., Amir D., Haas E., Fersht A.R. (2009). Time-resolved fluorescence resonance energy transfer study shows a compact denatured state of the B domain of protein A. Biochemistry.

[49] Huang F., Sato S., Sharpe T.D., Ying L., Fersht A.R. (2007). Distinguishing between cooperative and unimodal downhill protein folding. Proc. Natl. Acad. Sci. USA.

[50] Oikawa H., Suzuki Y., Saito M., Kamagata K., Arai M., Takahashi S. (2013). Microsecond dynamics of an unfolded protein by a line confocal tracking of single molecule fluorescence. Sci. Rep..

[51] Oikawa H., Kamagata K., Arai M., Takahashi S. (2015). Complexity of the folding transition of the B domain of protein A revealed by the high-speed tracking of single-molecule fluorescence time series. J. Phys. Chem. B.

[52] Rossow M.J., Sasaki J.M., Digman M.A., Gratton E. (2010). Raster image correlation spectroscopy in live cells. Nat. Protoc..

[53] Digman M.A., Caiolfa V.R., Zamai M., Gratton E. (2008). The phasor approach to fluorescence lifetime imaging analysis. Biophys. J..

[54] Cheng C.-H., Ishii K., Tahara T. (2017). RNA and DNA hairpin dynamics studied by temperature-controlled 2D fluorescence lifetime correlation spectroscopy. Proc. Asian Spectrosc. Conf..

[55] Otosu T., Yamaguchi S. (2018). Quantifying the diffusion of lipids in the proximal/distal leaflets of a supported lipid bilayer by two-dimensional fluorescence lifetime correlation spectroscopy. J. Phys. Chem. B.

[56] Castellana E.T., Cremer P.S. (2006). Solid supported lipid bilayers: From biophysical studies to sensor design. Surf. Sci. Rep..

[57] Allhusen J.S., Conboy J.C. (2017). The ins and outs of lipid flip-flop. Accounts Chem. Res..

[58] Zhang L.F., Granick S. (2005). Lipid diffusion compared in outer and inner leaflets of planar supported bilayers. J. Chem. Phys..

[59] Hetzer M., Heinz S., Grage S., Bayerl T.M. (1998). Asymmetric molecular friction in supported phospholipid bilayers revealed by NMR measurements of lipid diffusion. Langmuir.

[60] Schoch R.L., Barel I., Brown F.L.H., Haran G. (2018). Lipid diffusion in the distal and proximal leaflets of supported lipid bilayer membranes studied by single particle tracking. J. Chem. Phys..

